# Case Report: Late-onset primary hemophagocytic lymphohistiocytosis leading to the diagnosis of Griscelli syndrome type 2 in a young woman with phenotypically inapparent partial albinism

**DOI:** 10.3389/fimmu.2025.1604460

**Published:** 2025-08-07

**Authors:** Johanna Rausch, Stephanie Herold, Simone Liebhäuser, Yagmur Bülbül, Edite Antunes Ferreira, Till Wenz, Kevin Jan Legscha, Matthias Bros, Florian Butsch, Oliver Kriege, Klaus Warnatz, Miriam Groß, Kai Lehmberg, Helena Clara Lichtenfeld, Paul La Rosée, Markus Philipp Radsak, Matthias Theobald, Hakim Echchannaoui, Markus Munder

**Affiliations:** ^1^ Department of Hematology and Medical Oncology, University Medical Center, Johannes Gutenberg University, Mainz, Germany; ^2^ German Cancer Consortium (DKTK), Partner Site Frankfurt/Mainz, Mainz, Germany; ^3^ Department of Hematology and Medical Oncology, Johanniter Hospital Bonn, Bonn, Germany; ^4^ Department of Dermatology, University Medical Center, Johannes Gutenberg University, Mainz, Germany; ^5^ Center for Chronic Immunodeficiency, Faculty of Medicine, University of Freiburg, Freiburg, Germany; ^6^ Department of Rheumatology and Clinical Immunology, University Medical Center Freiburg, Faculty of Medicine, University of Freiburg, Freiburg, Germany; ^7^ Institute of Immunodeficiency, University Medical Center Freiburg, Faculty of Medicine, University of Freiburg, Freiburg, Germany; ^8^ Division of Pediatric Stem Cell Transplantation and Immunology, Clinic for Pediatric Hematology and Oncology, University Medical Center Hamburg-Eppendorf, Hamburg, Germany; ^9^ Department of Internal Medicine II, Schwarzwald-Baar-Klinikum, Villingen-Schwenningen, Germany; ^10^ Department of Hematology and Oncology, Donau-Isar Hospitals, Deggendorf, Germany; ^11^ Research Center for Immune Therapy [Forschungszentrum für Immuntherapie (FZI)], University Medical Center (UMC) of the Johannes Gutenberg University, Mainz, Germany; ^12^ Department of Hematology and Medical Oncology, Diakonissen-Stiftungs-Krankenhaus Speyer, Speyer, Germany

**Keywords:** Griscelli syndrome type 2, hemophagocytic lymphohistiocytosis, RAB27a variant, polymorphonuclear neutrophils, degranulation defect, hyperinflammation, case report

## Abstract

Griscelli syndrome type 2 (GS-2) is a rare congenital immune dysfunction characterized by partial albinism and recurrent episodes of hemophagocytic lymphohistiocytosis (HLH). It is caused by a variant in the gene encoding Rab27a leading to a degranulation defect in melanocytes, natural killer (NK)- and T cells. Prognosis of patients with GS-2 is limited by repetitive episodes of life-threatening HLH with onset in early childhood. The only curative treatment is an allogeneic hematopoietic stem cell transplantation (HSCT). Here, we report on an 18 year old female patient with a homozygous missense p.Arg50Glnfs*35 variant in exon 2 of *RAB27A* who presented with an exceptionally late onset of severe HLH. Her phenotypically inapparent albinism complicated to correctly diagnose GS-2. Immune function assays confirmed a T- and NK cell degranulation deficiency characteristic for patients with primary HLH, while microscopic hair analysis revealed melanin clumps secondary to melanocyte functional impairment. To understand why disease onset occurred unusually late in this patient, we investigated the patient’s T cell and polymorphonuclear neutrophil (PMN) function in more detail. We could show that intracellular granzyme B storage in cytotoxic T cells was increased compared to healthy donors and that the patient’s T cells maintained some degranulation activity. Both, antigen-specific cytotoxic response and proliferation capacity of the patient’s T cells were preserved. We demonstrate for the first time that also PMN degranulation, assessed as stimulation-induced CD66b and CD11b cell membrane expression, is dysfunctional in patients with Rab27a deficiency-associated primary HLH. The patient was treated with steroids and cyclosporine A for immunosuppression to control the HLH. After two severe episodes within only a few months, she eventually received an allogeneic HSCT and has not experienced further HLH episodes for now more than 3 years after the HSCT procedure. This case should raise awareness for the possibility of initial manifestation of primary, genetically-determined HLH even in adult patients.

## 1 Introduction

GS-2 is an inherited autosomal recessive immune disorder characterized by primary HLH and partial albinism usually with onset in early childhood (1, 2). It results from a variant in the gene encoding Rab27a, a protein of the GTPase family involved in vesicular transport and organelle dynamics. Rab27a is highly expressed in melanocytes and leukocytes (3). In melanocytes, Rab27a is involved in melanosome transport (4) explaining the partial albinism in case of loss-of-function variants, though rare cases without apparent albinism have been described (5–8). In cytotoxic T lymphocytes (CTLs), NK- and mast cells, Rab27a plays an important role in the secretion of cytolytic granules by interacting with the priming factor Munc13-4 (2, 3, 9) which is a member of the Unc13 protein family encoded by the gene *UNC13D* (Unc-13 Homolog D) (10). Variants in either protein disable the release of lytic granules at the immunologic synapse causing reduced cytotoxicity (11, 12). This leads to an insufficient elimination of infectious triggers with persistent immune stimulation with secondary systemic hyperinflammation and cytokine storm presenting as HLH (13, 14).

HLH is characterized as a syndrome of fever, splenomegaly and cytopenia sustained by a dysregulated proliferation and activation of T cells and macrophages (15, 16). Diagnostic markers are severely increased levels of serum ferritin and soluble interleukin-2 receptor (sIL2R), hypertriglyceridemia, hypofibrinogenemia, hemophagocytosis in the bone marrow and low NK cell degranulation (17). Additionally, inflammatory cytokines such as interferon-gamma (IFN-γ), tumor necrosis factor alpha (TNF-α), interleukin 1 (IL-1), IL-2, IL-6, IL-10, IL-12, IL-16 and macrophage colony stimulating factor (M-CSF) are hyper-secreted (18–22).

Primary HLH is associated with several inheritable gene defects, most prominently variants in *PRF1* (perforin 1), *UNC13D*, *STX11* (Syntaxin 11), *MUNC18-2* (also called *STXBP2*, Syntaxin Binding Protein 2) or *RAB27A* (13, 15, 16). All these variants interfere with proper immunity and provoke repetitive episodes of HLH, frequently with fatal outcome. Besides primary HLH, there are many known triggers causing secondary HLH independent of known monogenetic predisposition such as infections (e.g. Epstein-Barr virus, EBV), rheumatological disorders (e.g. Still’s disease or systemic lupus erythematosus), malignant disorders (e.g. lymphomas) or immune modifying therapy (e.g. stem cell transplantation or checkpoint inhibitors) (13, 16). First line treatment for patients with HLH is guided by the HLH-1994 protocol (15, 16) with modifications or additions, however HSCT remains the only curative therapy for patients with primary HLH (15, 23).

## 2 Case description

An 18-year-old woman presented at our emergency unit with fever, cough, epistaxis and temporary memory loss after several weeks of fatigue. She was in a severely reduced general condition with positive shock index and thus admitted to our ward with diagnosis of sepsis for further treatment. Physical examination showed a so far undiagnosed splenomegaly without other obvious phenotypical abnormalities. Laboratory results revealed pancytopenia (leukocytes: 930/µl, hemoglobin: 8.0 g/dl, thrombocytes: 17/nl), elevated transaminases (ALT 126 U/l [normal range <35 U/l], AST 139 U/l [5–31 U/l]), CRP (104 mg/l [< 5 mg/l]) and LDH (819 U/l [< 245 U/l]) as well as an undetectable haptoglobin (<0.08g/l [0.35-2.5 g/l]) and slightly elevated bilirubin (1.3 mg/dl [0.2-1.2 mg/dl]). Additional tests showed no blasts in the differential blood count, high ferritin (5712 ng/ml [5–200 ng/ml]), elevated triglycerides (407 mg/dl [< 150 mg/dl]), hypogammaglobulinemia (IgG 3.46g/l [5.5-16.3 g/l]) and elevated sIL-2R (6,073 Mio U/ml, >2400U/ml diagnostic for HLH/94.2 ng/ml, [1.9-13.1 ng/ml]). The patient history yielded one similar prior episode of fever with meningitis-like symptoms and diarrhea at the age of 4 years. Laboratory routine revealed a mild anemia and elevated CRP (74 mg/l [< 5 mg/l]), but no elevation of transaminases. A cerebrospinal fluid diagnostic excluded a meningitis and the patient was eventually discharged with the diagnosis of a gastroenteritis. She did not report of other recurrent fevers, muscle weakness, neurologic symptoms or a diagnosed cytopenia. The family was of Middle Eastern origin, and her parents were consanguineous, though the exact degree of relationship is unknown. Her father died of a glioblastoma, her mother and her two siblings were healthy.

**Figure 1:**
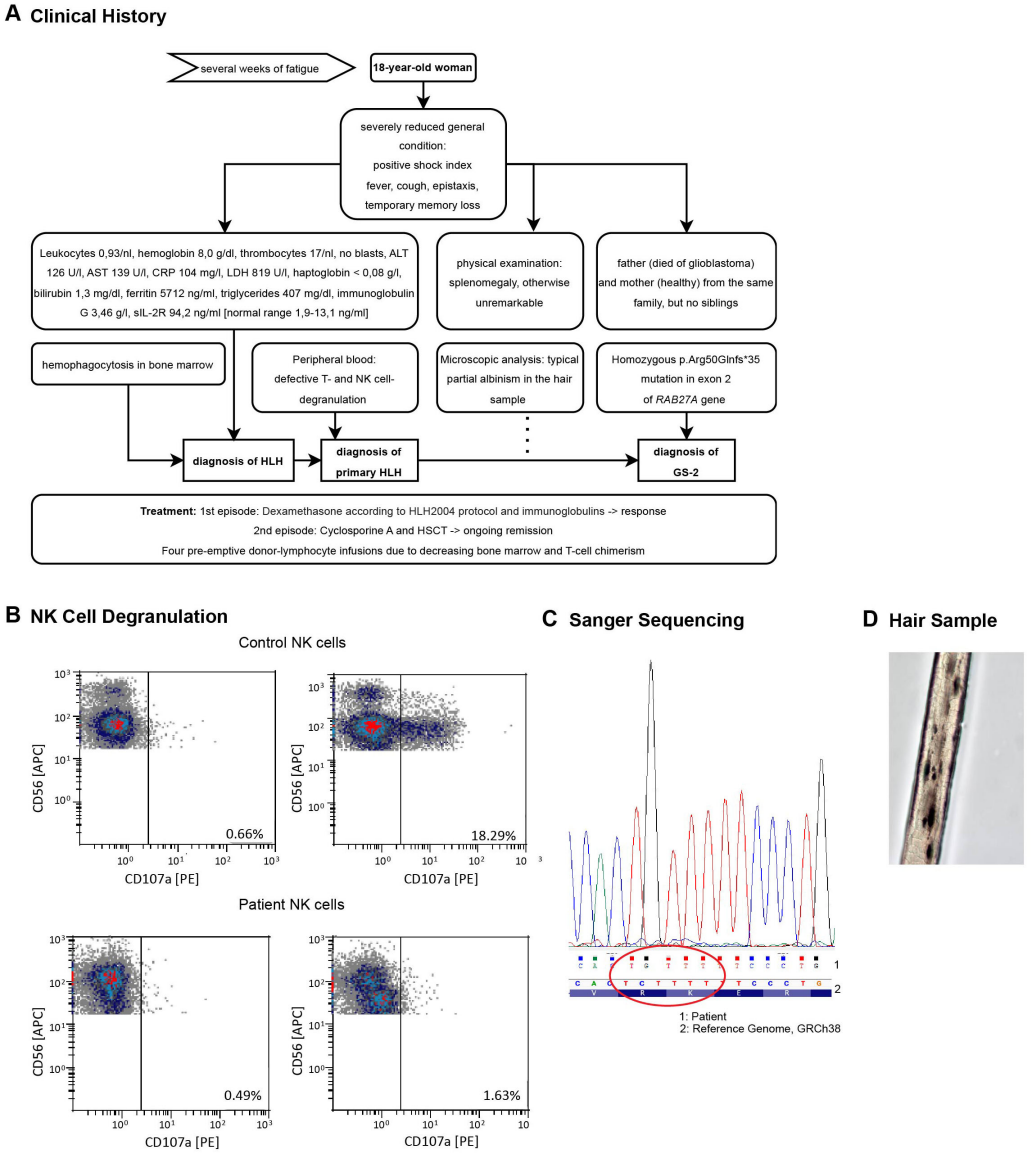
**(A)** Diagnostic workup leading to the diagnosis of GS-2. **(B)** Degranulation defect in patient’s NK cells. Cells were isolated from the peripheral blood during the first HLH episode. Degranulation was assessed by flow cytometry measuring the CD107a cell membrane expression. The top depicts HD samples, the bottom cells from the HLH patient. Results for NK cells are shown without (left) or after (right) *in vitro* stimulation with K562 cells for 2 hours. The data was kindly provided by the Center for Chronic Immunodeficiency of the Medical Center - University of Freiburg, Freiburg, Germany. **(C)** Sanger Sequencing results from the patient’s blood detecting the p.Arg50Glnfs*35 variant in exon 2. The results were displayed with Chromas software and aligned to the GRCh38 genome displayed by the Integrative Genomic Viewer (IGV) (Version 2.16.2). **(D)** Melanin clumps and partial albinism in a hair sample, light microscopy, 40X magnification.

Diagnostic workup (**Figure 1A**) demonstrated no signs of relevant viral or bacterial infection as well as no Adenovirus, Cytomegalovirus (CMV), EBV, Herpes simplex virus (HSV), Varicella-Zoster virus (VZV) as determined by PCR in serum samples. Serum PCR was weakly positive for human herpesvirus 6 (HHV-6) (5.7x10^2^ copies/ml) and seroconversion after SARS-CoV2 infection (IgM negative/IgG positive) approximately 6 months earlier. Computed tomography (CT) scan showed hepatosplenomegaly and anasarca but no evidence of lymphoma. Anamnestic, clinical and a broad serological work-up did not reveal any signs of autoimmune disease. We performed a bone marrow biopsy to rule out hematological malignancy. Erythropoiesis constituted more than 50% of the nucleated cells, whereas granulopoiesis was reduced but with normal cellular differentiation. We excluded leishmaniosis and mycobacterial infection by PCR of the bone marrow sample. Histologically, we observed signs of hemophagocytosis. Thus, the patient fulfilled 7/8 of the initial diagnostic criteria (fever, splenomegaly, pancytopenia, hypertriglyceridemia ≥ 265mg/dl, hemophagocytosis in bone marrow and hyperferritinemia ≥500 µg/l as well as elevated sIL-2R), for the diagnosis of HLH and intravenous admission of dexamethasone was consequently started according to HLH-2004 protocol (16). On day 3 after admission, we added intravenous immunoglobulins. Cytotoxic treatment with etoposide was omitted because of the patient’s young age and missing fertility-preserving measures.

Since no trigger factor for secondary HLH was apparent in our extensive work-up, we considered the possibility of primary HLH. NK cell function in the peripheral blood was evaluated by flow cytometry analysis of the degranulation marker CD107a (LAMP-1) and revealed defective activation-induced degranulation of NK cells in both, CD56^bright^ and CD56^dim^ populations (**Figure 1B**). This finding, complemented by the absence of infectious, rheumatological or malignant causes, led to the diagnosis of a primary HLH. Targeted gene sequencing demonstrated a homozygous p.Arg50Glnfs*35 variant (Del-Ins) in exon 2 of the *RAB27A* gene (**Figure 1C**), previously described in patients with GS-2 (24–26). Microscopic analysis of her hair revealed the typical picture of partial albinism with melanin clumping (**Figure 1D**), even though phenotypically no albinism was apparent. With proof of hereditary HLH, HSCT was indicated and the donor search process initiated.

**Figure 2:**
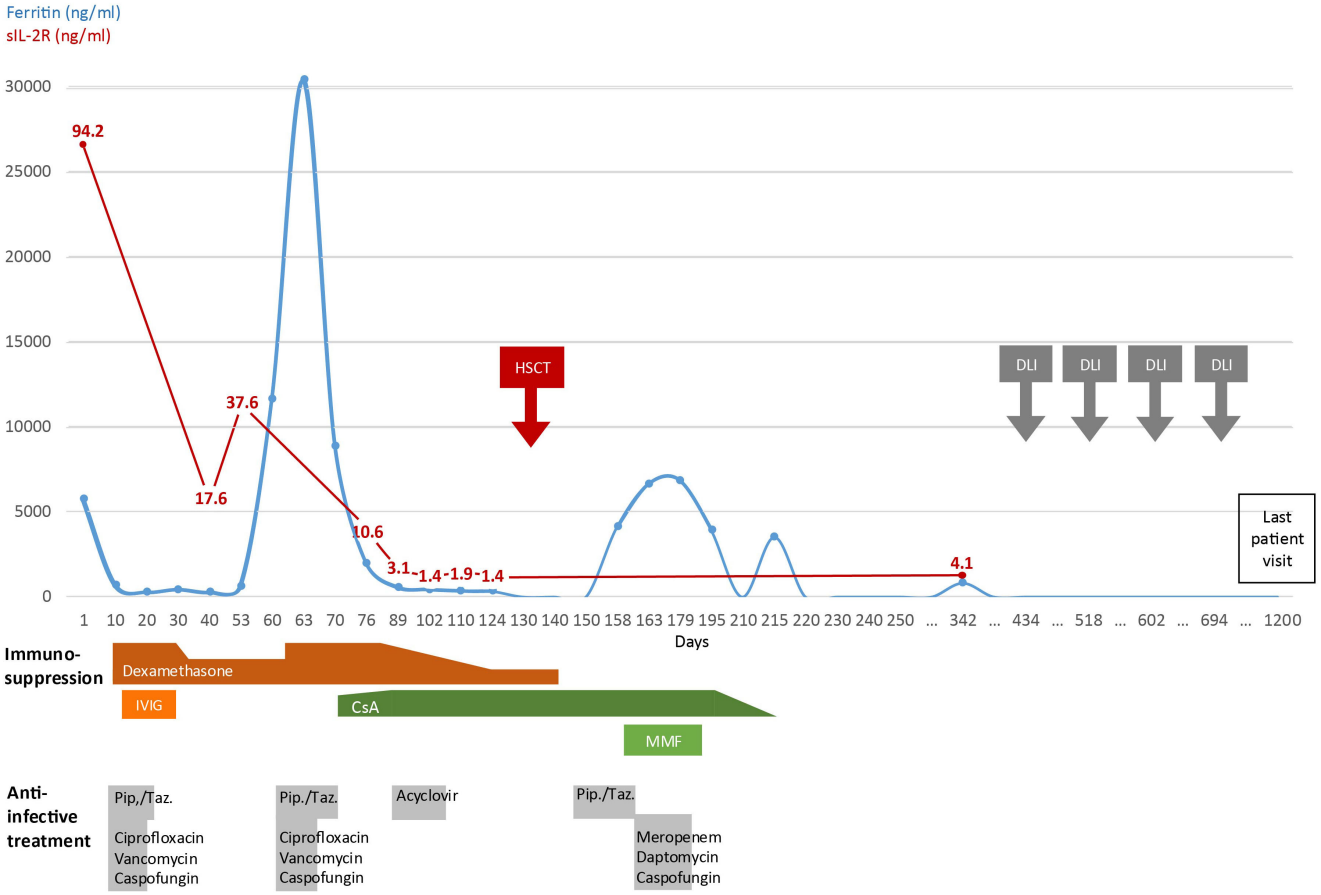
Timeline of clinical development. Disease activity displayed by serum ferritin and sIL-2R levels and administered therapy since first day of admission into hospital (day 1) until last patient visit. (IVIG, intravenous Immunoglobulin G; CsA, Cyclosporine A; MMF, mycophenolate mofetil; Pip./Taz., piperacillin-tazobactam; HSCT, hematopoietic stem cell transplantation; DLI, donor lymphocyte infusion).

The patient responded well to steroid therapy and was discharged on day 12 after admission to be further treated and monitored in the outpatient setting (**Figure 2**). One month later, she developed fever and a second episode of HLH while on 4 mg dexamethasone daily. Despite escalation of dexamethasone to 20 mg daily, she remained unresponsive with persistent pancytopenia and highly elevated ferritin levels (30464 ng/ml) (**Figure 2**). An immunosuppressive therapy with cyclosporine A was initiated (plasma target level of 200 ng/ml) as bridging to HSCT. After 18 days, the patient was discharged with improved general condition, decreased laboratory inflammation markers, reconstituted granulocytes and thrombocytes and reduced, but still elevated ferritin levels (4253 ng/ml). After oocyte cryopreservation, her pre-transplantation work-up disclosed normal, slightly hypocellular bone marrow (by cytology and histology), normal spleen size, but moderate hepatomegaly (midclavicular line 15 cm). The patient received an HSCT (conditioning: Fludarabine, Melphalan, Alemtuzumab) from an HLA-B-Mismatch unrelated donor 136 days after her first admission. She developed one grand-mal seizure in the context of a sepsis during neutropenia after conditioning chemotherapy. The microbiologic and pathologic workup remained without relevant findings, the cranial magnetic resonance imaging (cMRI) showed unspecific signal alterations in cortex in both hemispheres. In the follow up, radiologic findings normalized, the patient remained fully asymptomatic and anticonvulsive therapy was terminated on day 390. Three months (day 221) after HSCT she developed a late-onset acute Graft-versus-host disease (GvHD) of the skin that fully vanished after one week of local corticosteroids. Addressing a decreasing bone marrow and T cell chimerism, she received four pre-emptive donor-lymphocyte infusions (DLI) - 10, 13, 15 and 19 months after HSCT - without developing consecutive GvHD. Latest determination of chimerism in the bone marrow was 99.8% for CD15^+^ granulocytes, and 98.4% for CD3^+^ T cells. The patient has remained in remission to date with no further HLH episodes.

To decipher why the first HLH episode as a sign of an underlying immune dysfunction occurred unusually late in this patient, we investigated several key cellular immune functions *in vitro*.

**Figure 3:**
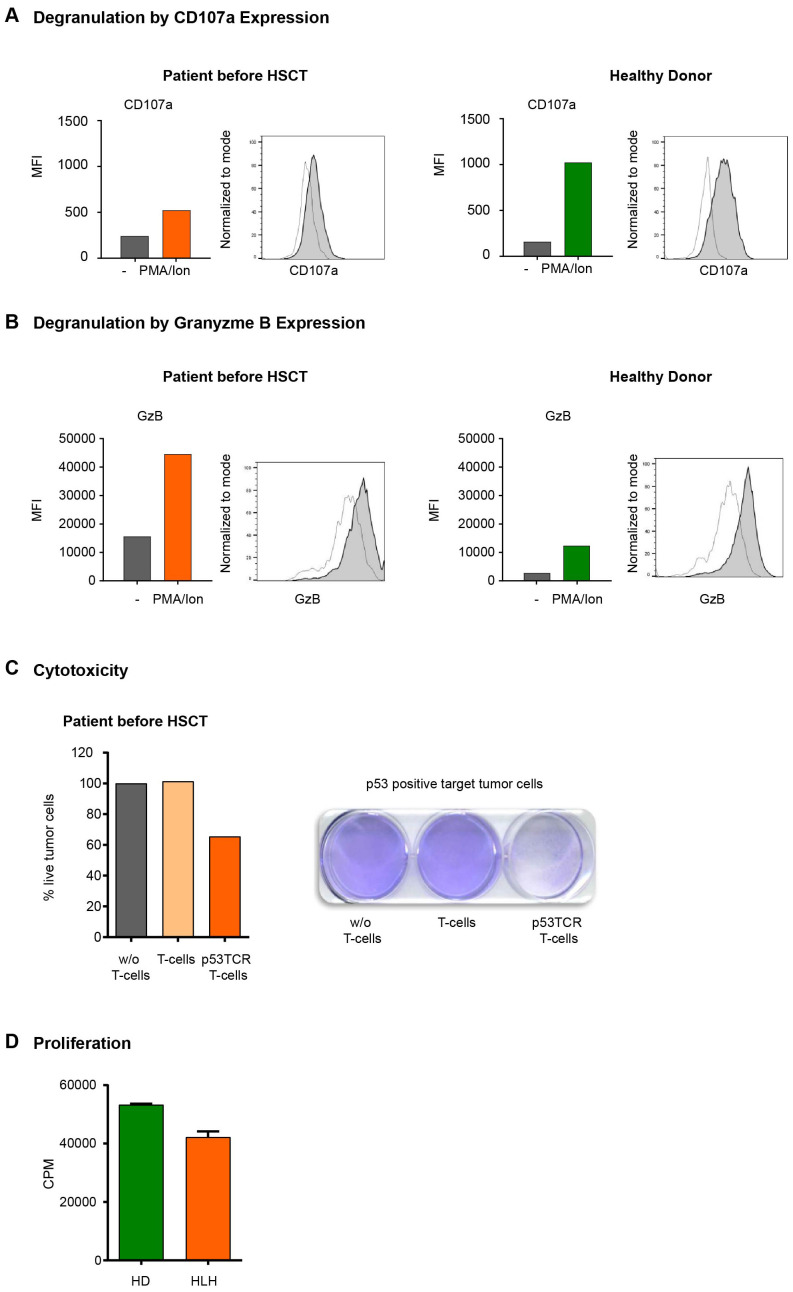
**(A, B)** Cell-membrane CD107a **(A)** and intracellular Granzyme B **(B)** expression in T cells. **(A)** Flow cytometry expression analysis of the degranulation marker CD107a in CD3+ T cells from HD and HLH patient detected as MFI values of CD107a expression in HD T cells and HLH patient (unstimulated *vs* stimulated). Medium only (transparent histogram) or stimulated with PMA/Ionomycin (gray histogram). **(B)** Analysis of intracellular granzyme B (GrB) expression in CD3+ T cells from healthy control donor and HLH patient. MFI values of GrB expression in HD T cells and HLH patient (unstimulated *vs* stimulated). Medium only (transparent histogram) or stimulated with PMA/Ionomycin (gray histogram). **(C)** Cytotoxic activity of CD3+ T cells. The cytolytic activity of the HLH patient’s T cells was assessed in tumor colony-forming assays after transduction with a p53(264-272) specific TCR. Tumor cells (Saos2/143) were co-cultured for 24h either without (w/o) T cells, with unmodified T cells or with p53(264-272) TCR transduced T cells. After repetitive washing steps only viable tumor cells remain attached to the cell culture plate and are visualized by staining with crystal violet dye and measured by optical density (OD) of the dye. Primary data and OD quantification with normalization (100% viability in the condition w/o T cells) of this experiment are demonstrated. **(D)** Proliferation of CD3+ T cells from HD and HLH patient as determined by 3H-Thymidine assay.

In addition to the impaired degranulation observed in NK cells, we extended the analysis to T cells. Interestingly, the patient’s CD3^+^ T cells showed a clear reduction rather than a complete defect in degranulation (measured as CD107a expression by flow cytometry upon stimulation (with PMA/Ionomycin) compared to healthy donors (HDs) (**Figure 3A**). Moreover, flow cytometry revealed an increased intracellular granzyme B (GrB) concentration compared to a HD independent of the stimulation with PMA (**Figure 3B**).

We next analyzed to which degree the reduced degranulation would affect T cell-mediated cytotoxicity. For this, we used our well-established model of peptide antigen specific CD3^+^-mediated cellular cytotoxicity (27). Briefly, peripheral blood mononuclear cells (PBMC) of the patient were retrovirally transduced with a T cell receptor (TCR) with specificity for the HLA-A2.1 restricted p53(264-272) peptide. After 4 days of peptide specific stimulation the cytolytic function of p53TCR-modified CD3^+^ T cells from the patient was assessed in overnight coculture with a target tumor cell line (osteosarcoma Saos2/143) expressing the p53(264-272) peptide (**Figure 3C**). Importantly, the patient’s TCR-expressing CD3^+^ T cells induced relevant antigen-specific tumor cytotoxicity compared to non-transduced T cells. Next, we measured the proliferation capacity of the patient’s T cells, following our established methodology (28). CD3^+^ T cells were isolated from peripheral blood and stimulated with agonistic anti-CD3/CD28-beads as described (29). Proliferation was determined by [^3^H]-thymidine pulsing as described before (28). Here we demonstrated that the proliferation capacity of the patient’s CD3^+^ T cells was in the same range as the corresponding HD controls (**Figure 3D**).

Expression of Rab27a in PMN has previously been demonstrated (30, 31) and its involvement in PMN tertiary and specific granule mobilization was shown (32). This latter finding was generated by sophisticated blocking strategies in normal donor PMN. We had the unique opportunity to analyze *in vitro* degranulation efficacy in *RAB27A* mutated (and therefore potential loss-of-function) PMN from our patient, before and after HSCT.

**Figure 4:**
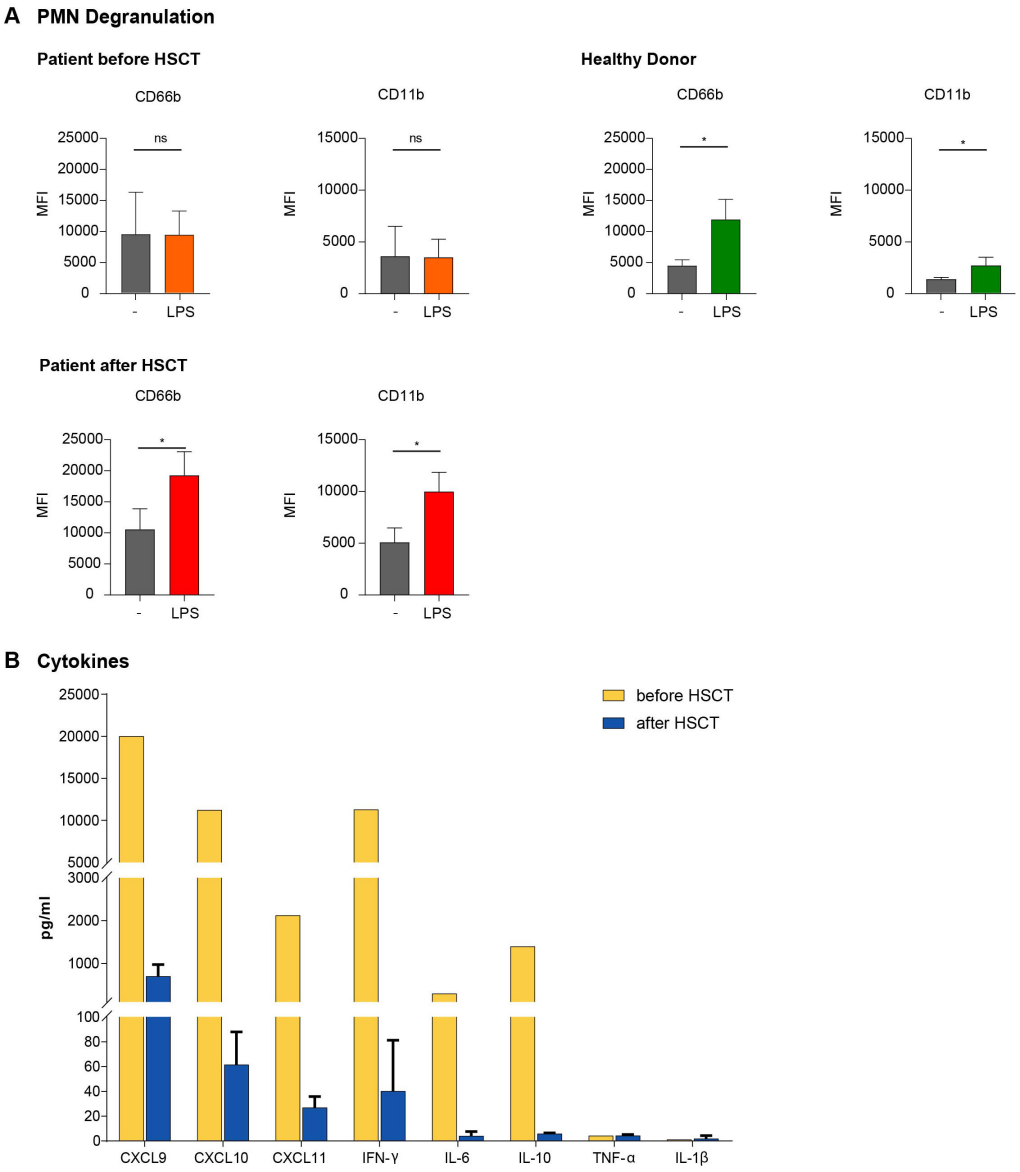
**(A)** Defective PMN Degranulation activity in GS-2. PMN of HD and the GS-2 patient were stimulated with LPS for 90 minutes. Expression of the surface markers CD66b and CD11b was assessed by flow cytometry (MFI with mean ± SEM) as correlate for the extent of degranulation/activation. n=5 samples from different HD, n=3 samples from the GS-2 patient acquired at different time points prior to and after HSCT (month 5, 6, 7), respectively. Statistical calculations were performed with Welch´s t test (*p <0.05; ns, not significant) with unstimulated control as reference. **(B)** HLH-associated cytokine elevations in the peripheral blood of the GS-2 patient Cytokine levels were assessed during the second HLH episode and at three different time points post-transplantation (months 5, 6, 7). All samples were taken without concurrent infection, fever or immunosuppresive medication. We compared pre-transplant HLH-associated (yellow) with the mean of post-transplant (blue) cytokine levels.

Degranulation of certain PMN granule subtypes can be quantified by cell membrane incorporation of granule-localized membranous proteins, which become externalized and are present in the cell membrane during the process of degranulation. Activation–induced upregulation of CD66b and CD11b is associated with degranulation of tertiary and specific PMN granules (32, 33). Upon lipopolysaccharide (LPS)-mediated activation we monitored cell membrane CD11b and CD66b expression by flow cytometry. In contrast to HD, our patient did not show a significant activation-induced increase in CD11b and CD66b expression, demonstrating a severe degranulation deficiency of her PMNs. Similar analysis was performed after HSCT, demonstrating a normal degranulation capacity of the allogeneic PMNs, correlating to the clinical remission of the patient (**Figure 4A**).

Finally, we determined a broad array of inflammatory cytokines and chemokines by Bead Array technology (27) in the serum of the patient. The serum samples were collected during the second hyperinflammatory HLH episode as well as after HSCT in the absence of clinically apparent inflammatory problems or immunosuppressive medication. Cytokine levels were assessed prior to stem cell transplantation during the second HLH episode and post transplantation. As expected, IFN-γ (11290 *vs*. 28pg/ml), IL-6 (293 *vs*. 4 pg/ml) and IL-10 (1395 *vs*. 6 pg/ml) were significantly increased during the HLH episode. We did not detect changes in TNF-α or IL-1β levels, but observed an increase of several chemokines, especially of CXCL9 (>20000 [above highest standard] *vs*. 374 pg/ml), CXCL10 (11235 *vs*. 61 pg/ml) and CXCL11 (2125 *vs* 27 pg/ml), all ligands of the chemokine receptor CXCR3 (**Figure 4B**).

## 3 Discussion

We report a case of exceptionally late onset of GS-2 in a patient with a homozygous missense p.Arg50Glnfs*35 variant in exon 2 of *RAB27A*. Phenotypically, partial albinism was inapparent, however a hair sample confirmed partial albinism with melanin clumps. Several reports of patients with GS-2 sine albinism exist (6–8). One retrospective study based on data of the International HLH Registry of the Familial hemophagocytic lymphohistiocytosis (FHL) study group from 1989 to 2013 identified six cases of GS-2 without albinism (8). These patients had biallelic variants in *RAB27A* (localization A76, R141, Y159, and S163) that altered the binding site with Munc13–4 but not melanophilin (MLPH). The disruption of Rab27a/Munc13-4-binding disabled the secretion of cytolytic granules in leukocytes while normal skin pigmentation was maintained as reflected by intact melanophilin binding (8). In a different report, a novel *RAB27A* gene variant (Val143Ala) was discovered in a patient without formation of typical melanin clumps in his hair. Here again, Rab27a/MLPH binding was preserved (5). The homozygous p.Arg50Glnfs*35 Rab27a variant in our patient is usually associated to albinism (34–36). Albeit our patient showed no phenotypical signs of albinism, her hair analyses revealed partial melanin clumping. Consequently, physicians should not be misled by phenotypic appearance, since subtle melanin abnormalities might not be apparent macroscopically.

Exceptional about this case was the unusually late onset of the first life-threatening HLH episode at 18 years of age. Late manifestations of primary HLH are rare but genetic alterations should still be considered as a potential underlying cause (37). A different case report with first manifestation at the age of 29 years reported a novel GATA2 variant and, like in our case, a HSCT was conducted for curative treatment (38). In patients with *RAB27A* variant, a similar finding was reported in a case report of a 24-year-old female with a heterozygous variant presenting with neurologic symptoms and albinism (24). The patient carried a c.259G > C; p.Ala87Pro missense variant in exon 4 and importantly also a c.149delG; p.Arg50Glnfs*35 variant in exon 2 identical to the homozygous variant in our patient. A recent case report described a novel homozygous *RAB27A* c.551G > A p.(R184Q) mutated patient without albinism and an onset of disease at 35 years and concurrent EBV infection (39). Possible reasons why symptoms arise after adolescence in some patients even with homozygous variants remain incompletely understood. The timing of symptom onset might be influenced by external or internal triggering events, such as infections which may exacerbate the underlying molecular defect (40–42). This is supported by a murine model with a *MUNC13–4* deficiency (responsible for FHL 3), in which an infectious trigger (lymphocytic choriomeningitis virus) was necessary for the development of an HLH (43). Our patient had no concurrent infection but rather a history of infection with SARS-CoV six months ago which has been associated with the development of secondary HLH (44–46). The negative IgG-levels for EBV, CMV, HSV and VZV suggest an infection naïve patient history during childhood and adolescence. The residual protein expression from the gene variant might have delayed disease manifestation as previously discussed (47). Unfortunately, Rab27a protein expression levels were not tested and these data can therefore not be provided. Alternatively, we speculate that a compensation by other functional molecules or activation of alternative signaling pathways might mitigate the impact of the variant Rab27a protein for a time, postponing clinical symptoms. The analysis of our patient’s T cell function suggests a preserved low-level degranulation capacity (**Figures 3A, B**), an intact proliferation capacity (**Figure 3D**) and a functioning antigen-specific cytotoxic response at least *in vitro* (**Figure 3C**), possibly contributing to a late occurrence of HLH episodes. Impaired T cell cytotoxicity had been described earlier in a murine model of Rab27 deficiency (48). However, this deficient cytotoxicity was apparent towards FAS-negative target cells, while in our assay system, the Fas-FasL cytotoxic pathway is present and has likely contributed (besides the partial T cell degranulation capacity) to the preserved T cell cytotoxicity. The intracellular storage of GrB was higher than in the healthy donor sample as described before (49), either as a coping strategy or as reaction to a prolonged antigen exposure due to the insufficient antigen clearance (12, 39). Whether the remaining activity of CTLs had been sufficient to fight pathogens and prevent hyperinflammation remains unclear.

Defective NK- and T cell degranulation is a recognized key factor of GS-2. Novel about our case is the analysis of PMN degranulation in primary patient material as part of the diagnostic workup. Previous studies suggested that *RAB27A* deficient PMN exhibit an impaired myeloperoxidase (MPO) or matrix metalloproteinase 9 (MMP-9) exocytosis upon granulocyte macrophage colony-stimulating factor (GM-CSF) or LPS stimulation in murine models (31, 50). Similarly, *RAB27A*-downregulation (31) or Rab27a inhibition (32) lead to reduced tertiary and specific granule mobilization in human PMNs. In line with these results, we demonstrate for the first time that mobilization of gelatinase granules (CD11b) and azurophilic granules (CD66b) upon LPS stimulation is indeed defective in PMN of patients with GS-2 (**Figure 4A**). Interestingly, these results in primary human PMN partially are in contrast to data from the murine *RAB27A* knockout model, which largely suggest Rab27a-independent up-regulation of CD11b in neutrophils, yet upon GM-CSF stimulation (50).

In line with previous reports, we observed high levels of IFN-γ, IL-10, IL-6 and of the CXCR3 ligands CXCL9, CXCL10 and CXCL11 in the peripheral blood during active HLH (51–54). Especially elevated IFN-γ and IL-10 levels have been shown to be characteristic of HLH with high sensitivity and specificity (20, 51, 54). Emapalumab, an IFN-γ-blocking antibody, was successfully tested in a phase 2–3 study in children with primary HLH and is now available as specific cytokine-directed therapy for future therapeutic approaches in patients with primary HLH (18, 52). CXCL9, CXCL10 and CXCL11 are IFN-γ-inducible ligands of CXCR3, which functions as an inflammatory chemokine receptor on CD4+ Th1, CD8+ CTL, NK and dendritic cells (DC) (53) and is upregulated after DC-mediated T cell activation. Together, the high levels of these cytokines in our patient are consistent within the setting of HLH.

In summary, we present a case of GS-2 with unusually late onset and clinical phenotype without remarkable or suggestive features. We demonstrate completely absent NK- and PMN-degranulation, but partially preserved T cell degranulation. The T cells remain functional with regard to their capacity to proliferate and to mount antigen-specific cytotoxicity. The reason why the patient remained asymptomatic throughout adolescence is unexplained. Our case report emphasizes the importance to consider genetic testing for primary HLH in adult patients with causally unclear HLH.

## 4 Methods

### 4.1 Isolation of T cells and intracellular granzyme B/surface CD107a expression

Peripheral blood mononuclear cells (PBMCs) from healthy control donor (HD) and HLH patient were isolated by Ficoll density gradient centrifugation. CD3 positive T cells were isolated with the EasySep™ Human T cell Enrichment Kit (Stemcell Technologies, Vancouver, Canada) and kept overnight in RPMI 1640 + 10% human AB Serum, 1% P/S and 1% L-Glutamine. To detect the surface membrane expression of the lysosomal-associated membrane protein 1 (LAMP1/CD107a) (as a surrogate marker for degranulation) and the intracellular granzyme B (GrB), CD3+ T cells were stimulated with Phorbol 12-myristate 13-acetate (PMA) + ionomycin (Sigma-Aldrich) for 5h in the presence of monensin (eBioscience) as described earlier (27, 29). CD107a surface expression and intracellular GrB expression were analyzed by flow cytometry and quantitated by showing mean fluorescence intensity (MFI).

### 4.2 NK cell degranulation

NK degranulation assays were performed as described in Bryceson et al. (2012) (55).

### 4.3 Proliferation assay

CD3 positive T cells isolated from the PBMCs of HD and the HLH patient were stimulated with agonistic anti-CD3/CD28-beads in the presence of IL-2 for 5–6 days (27) and T cell proliferation was assessed by the incorporation of [^3^H]thymidine as described before (28).

### 4.4 Cytotoxic assay

The cytolytic activity of the HLH patient’s CD3+ T cells was assessed in tumor colony-forming assay (CFA) after retroviral transduction with a p53(264-272) specific T cell receptor, as described (27). Briefly, effector T cells were co-cultured with antigen^+^ (Saos2/143) target tumor cells in 6-well plates in 37°C with 5% CO_2_ at an effector-to-target (E:T) ratio of 2:1. After 24h, T cells as well as non-adherent lysed tumor cells were washed out and the remaining adherent viable tumor cells were fixed (4% PFA) and stained with 0.5% crystal violet dye (Merck KGaA, Germany). Crystal violet was washed off by adding PBS and the plate scanned for visual evaluation of colony counts. For quantitative analysis, the dye was dissolved by adding 5% SDS and the corresponding optical density (absorbance) measured at 570nm using a microplate reader (Dynex MRX, Magellan BioScience), and values expressed as percent of tumor viability (27, 29).

### 4.5 Degranulation of PMN

Degranulation of PMN granule subtypes can be quantified by cell membrane incorporation of granule-localized membranous proteins, e.g. by activation–induced upregulation of CD66b and CD11b (32, 33). PMN of healthy donors and the HLH patient were stimulated with lipopolysaccharide (LPS) for 90 minutes. The surface expression of the markers CD11b and CD66b was then assessed by flow cytometry measuring the MFI (mean ± SEM). Statistical calculations were performed with Welch´s t test (*p <0.05) with unstimulated control as reference, n=5 from different healthy donors, n=3 from the HLH patient acquired at different time points prior and after HSCT.

### 4.6 Cytokines/chemokines

Secreted cytokines/chemokines *in vitro* culture and in serum were determined by Cytometric Bead Array (BD Biosciences, Franklin Lakes, NJ) according to the manufacturer protocol. Cytokine levels were assessed at one time point during the second HLH episode and at three different time points (months 5, 6, 7 post-transplantation). All samples were taken without concurrent infection or fever.

## Data Availability

The original contributions presented in the study are included in the article/supplementary material. Further inquiries can be directed to the corresponding authors.
